# A case series of hypercalcemia associated with pembrolizumab therapy: evidence for a calcitriol-mediated mechanism

**DOI:** 10.1210/jcemcr/luag042

**Published:** 2026-03-19

**Authors:** Victoria Belcher, Nadia Barghouthi, Sarah Hadique, Hania Woomer, Zeriel Wong, Adnan Haider

**Affiliations:** Department of Medicine, West Virginia University School of Medicine, Morgantown, WV 26505, USA; Division of Endocrinology and Metabolism, Department of Medicine, West Virginia University School of Medicine, Morgantown, WV 26505, USA; Division of Pulmonary, Critical Care, and Sleep Medicine, Department of Medicine, West Virginia University School of Medicine, Morgantown, WV 26505, USA; Department of Medicine, West Virginia University School of Medicine, Morgantown, WV 26505, USA; West Virginia University School of Medicine, Morgantown, WV 26505, USA; Division of Endocrinology and Metabolism, Department of Medicine, West Virginia University School of Medicine, Morgantown, WV 26505, USA

**Keywords:** hypercalcemia, pembrolizumab, calcitriol, immune checkpoint inhibitor

## Abstract

Immune checkpoint inhibitors enhance antitumor immunologic activity by blocking interaction between inhibitory immune checkpoint receptors. Pembrolizumab blocks the programmed cell death (PD-1) receptor and has been effective in the treatment of many malignancies. While many immune-related endocrinologic adverse effects have been described, immunotherapy-induced hypercalcemia is a rare adverse effect. We present 2 cases of patients who developed pembrolizumab-induced hypercalcemia. Both patients achieved resolution of hypercalcemia with discontinuation of pembrolizumab along with treatment with intravenous fluids, calcitonin, anti-resorptive therapy with either bisphosphonate or denosumab, and glucocorticoid therapy. These cases demonstrate the importance of recognizing immune checkpoint inhibitors as potential causes of parathyroid hormone (PTH)-independent hypercalcemia.

## Introduction

Immune checkpoint inhibitors (ICIs) have revolutionized cancer therapy by enhancing antitumor immunologic activity. ICIs work by blocking interaction between inhibitory immune checkpoint receptors such as programmed cell death-1 (PD-1) and cytotoxic T-lymphocyte–associated antigen 4 (CTLA-4) [1, 2]. Pembrolizumab, a monoclonal antibody targeting PD-1, has demonstrated efficacy across multiple malignancies but is frequently associated with immune-related adverse events (irAEs) resulting from loss of immune tolerance [3].

Endocrine irAEs typically manifest as thyroid dysfunction, hypophysitis, or insulin-deficient diabetes mellitus [4]. Hypercalcemia represents a rare metabolic complication, with only isolated cases described in association with ICI therapy [5]. The underlying mechanisms remain incompletely understood and may involve ectopic secretion of parathyroid hormone–related peptide (PTHrP), cytokine-mediated granulomatous inflammation, or other immune-mediated processes [6].

We describe 2 cases of pembrolizumab-induced hypercalcemia, highlighting the diagnostic complexity and therapeutic considerations of this rare disorder. Recognition of ICI-associated hypercalcemia is essential to enable prompt evaluation, facilitate safe continuation of immunotherapy, and expand our understanding of the spectrum of endocrine complications related to checkpoint blockade.

## Case presentation

### Case 1

A 65-year-old man with remote history of sarcoidosis and renal cell carcinoma status post left radical nephrectomy presented with an albumin-corrected calcium of 15.2 mg/dL (SI: 3.8 mmol/L) (reference range, 8.6-10.3 mg/dL [SI: 2.14-2.57 mmol/L]). Treatment with pembrolizumab had been initiated 3 weeks prior to presentation. He reported generalized weakness but denied other symptoms. Physical examination was unremarkable.

He was treated with intravenous fluids, calcitonin, and bisphosphonate therapy and was discharged 3 days later after improvement of corrected calcium levels to 10.9 mg/dL (SI: 2.7 mmol/L). Two weeks later, he was readmitted with hypercalcemia (corrected calcium 13.46 mg/dL [SI: 3.36 mmol/L]; ionized calcium 6.72 mg/dL [SI: 1.68 mmol/L], reference range 4.6-5.32 mg/dL [SI: 1.15-1.33 mmol/L]) and acute kidney injury (creatinine 2.47 mg/dL [SI: 218 μmol/L]) (reference range, 0.75-1.35 mg/dL [SI: 0.0663-0.1193 μmol/L]). His wife reported development of intermittent confusion.

### Case 2

An 83-year-old man with history of invasive urothelial carcinoma with recurrence on pembrolizumab for the past 2 years, ICI-associated arthritis on methotrexate (MTX), high-grade transitional cell carcinoma status post left nephrectomy, Merkel cell carcinoma, and stage 3 chronic kidney disease presented with generalized weakness and flu-like symptoms. Physical examination was unremarkable.

## Diagnostic assessment

### Case 1

Workup during the first admission was significant for parathyroid hormone (PTH) 7.4 pg/mL (SI: 0.78 pmol/L) (reference range, 8.5-77 pg/mL [SI: 0.89-8.09 pmol/L]), 25-hydroxyvitamin D (25-OH vitamin D) 77.8 ng/mL (SI: 194 nmol/L) (reference range, 30-100 ng/mL [SI: 75-250 nmol/L]), creatinine 1.8 mg/dL (SI: 159 μmol/L) which was at baseline, and phosphorus 2.5 mg/dL (SI: 0.81 mmol/L) (reference range, 2.3- 4.0 mg/dL [SI: 0.8065-1.2903 mmol/L]).

Given recurrent hypercalcemia, further evaluation was pursued on the second admission (Table 1). The patient had received 1 dose of pembrolizumab approximately 1 month prior. He denied use of calcium or vitamin D supplementation, thiazide diuretics, or lithium.

**Table 1:** Laboratory evaluation

Lab	Case 1	Case 2	Reference range
Albumin-corrected serum calcium	13.1 mg/dL (3.27 mmol/L)	13.8 mg/dL (3.44 mmol/L)	8.6-10.3 mg/dL (2.14-2.57 mmol/L)
Ionized calcium	6.72 mg/dL (1.68 mmol/L)	NA	4.60-5.32 mg/dL (1.15-1.33 mmol/L)
PTH	12.8 pg/mL (1.34 pmol/L)	8.8 pg/mL (0.92 pmol/L)	8.5-77 pg/mL (0.89-8.09 pmol/L)
AM cortisol	8.2 μg/dL (226 nmol/L)	18.6 μg/dL (513 nmol/L)	7.0-25.0 μg/dL (193-690 nmol/L)
25 Vitamin D	77.8 ng/mL (194 nmol/L)	67.4 ng/mL (168 nmol/L)	30-100 ng/mL (75-250 nmol/L)
1,25 Vitamin D	40 pg/mL (96 pmol/L)	132 pg/mL (317 pmol/L)	18-72 pg/mL (43-173 pmol/L)
Vitamin A	77 μg/dL (2.69 µmol/L)	45 μg/dL (1.57 µmol/L)	38-98 μg/dL (1.33-3.42 µmol/L)
ALP	90 U/L (1.50 µkat/L)	54 U/L (0.90 µkat/L)	45-115 U/L (0.75-1.92 µkat/L)
ACE	21 U/L (0.35 µkat/L)	NA	9-67 U/L (0.15-1.12 µkat/L)
Phosphate	2.7 mg/dL (0.87 mmol/L)	4.6 mg/dL 1.4(mmol/L)	2.3-4.6 mg/dL (0.74-1.4 mmol/L)
Creatinine	2.47 mg/dL (218 μmol/L)	2.63 mg/dL (233 μmol/L)	0.75-1.35 mg/dL (66-119 μmol/L)
Estimated GFR	29 mL/min/1.73m^2^	23 mL/min/1.73m^2^	≥60 mL/min/1.73m^2^
C-PTHrP	10 pg/mL (2.42 pmol/L)	14 pg/mL (3.4 pmol/L)	11-20 pg/mL (2.7-4.8 pmol/L)

Abnormal values are shown in bold font. Values in parenthesis are International System of Units (SI).

Abbreviations: ACE, angiotensin converting enzyme; ALP, alkaline phosphatase; C-PTHrP, C-terminal parathyroid hormone–related peptide; GFR, glomerular filtration rate; PTH, parathyroid hormone.

Given his remote history of sarcoidosis, cross-sectional imaging of the chest, abdomen, and pelvis without contrast was obtained. This showed a known 7-mm right lower lobe pulmonary nodule and calcifications consistent with prior granulomatous disease. No bony lytic lesions were identified to suggest metastatic disease. Imaging was reviewed by pulmonology with these findings determined to be more consistent with remote histoplasmosis rather than active granulomatous disease.

Given the suppressed PTH with inappropriately normal 1,25-dihydroxyvitamin D (calcitriol), the etiology of hypercalcemia was presumed to be pembrolizumab-induced 1,25-dihydroxyvitamin D–mediated hypercalcemia.

### Case 2

Initial evaluation revealed a calcium of 13.8 mg/dL (SI: 3.44 mmol/L). Cross-sectional imaging of the chest demonstrated diffuse bilateral ground-glass opacities and multiple subpleural calcified nodules. Pulmonology was consulted; however, given the patient's clinical improvement, bronchoscopy was deferred. Additional infectious workup, including *Histoplasma* and *Blastomyces* urine antigen testing, was negative.

Laboratory studies (Table 1) showed low-normal PTH, elevated 1,25-dihydroxyvitamin D, and normal 25-OH vitamin D, parathyroid hormone–related peptide (PTHrP), and alkaline phosphatase (ALP). There was no radiographic evidence of bony metastasis.

These findings collectively argued against hypercalcemia of malignancy and suggested a 1,25-dihydroxyvitamin D–mediated process. Given the absence of other causes, the etiology was presumed to be pembrolizumab-induced 1,25-dihydroxyvitamin D–mediated hypercalcemia.

## Treatment

### Case 1

Resolution of hypercalcemia was achieved with intravenous fluid volume expansion, calcitonin, and 1 dose of denosumab. Two weeks later, calcium trended upward and the patient then received 1 dose of intravenous zoledronic acid. Following evaluation by endocrinology and oncology, pembrolizumab was permanently discontinued to prevent recurrence of hypercalcemia. Prednisone 20 mg daily for 30 days was initiated approximately 1 month after his last dose of pembrolizumab.

### Case 2

The patient presented with elevated calcium following the 27th cycle of pembrolizumab. He was treated with intravenous fluids, calcitonin, and 1 dose intravenous furosemide to avoid volume overload. Pamidronate 60 mg was administered slowly following volume expansion. Additional bisphosphonate therapy was avoided due to chronic kidney disease and a solitary kidney. An oral prednisone taper was initiated at 100 mg daily for 5 days, followed by weekly dose reductions of 10 to 10 mg daily. After multidisciplinary discussion, pembrolizumab was discontinued.

## Outcome and follow-up

### Case 1

Calcium normalized within 18 days of initiating glucocorticoid therapy. Serum 1,25-dihydroxyvitamin D declined from 35 pg/mL (SI: 91 pmol/L) to 10 pg/mL (SI: 24.96 pmol/L) (reference range, 18-72 pg/mL [SI: 43-173 pmol/L]) by week 3. Prednisone was successfully discontinued at week 4.

### Case 2

Calcium normalized 2 weeks after initiating glucocorticoid therapy. One month later, mild hypocalcemia was noted and attributed to the pamidronate infusion. His prednisone taper was continued. Three months later, he was normocalcemic with a 1,25-dihydroxyvitamin D of 25 pg/mL (SI: 60 pmol/L). The patient successfully discontinued prednisone at month 4.

Both patients have maintained normal calcium levels and have remained off pembrolizumab.

## Discussion

Pembrolizumab, a PD-1 inhibitor, is associated with irAEs, including pneumonitis and, rarely, 1,25-dihydroxyvitamin D–mediated hypercalcemia [7]. Although humoral hypercalcemia of malignancy remains an important consideration in patients with a history of cancer, the presence of elevated 1,25-dihydroxyvitamin D supports a 1,25-dihydroxyvitamin D–mediated mechanism [8, 9]. We present 2 patients with pembrolizumab-induced hypercalcemia who responded to cessation of pembrolizumab and initiation of high-dose corticosteroids. Calcium levels remained normal after successful steroid tapering and discontinuation of pembrolizumab. Although the pathophysiology of pembrolizumab-mediated hypercalcemia is not fully understood, several mechanisms have been proposed.

Respiratory epithelial cells can convert inactive vitamin D into its active form through expression of 1α-hydroxylase, the enzyme responsible for converting 25-OH vitamin D into 1,25-dihydroxyvitamin D [10]. While 1,25 hydroxylation of vitamin D classically occurs in the proximal renal tubules, extrarenal expression has been demonstrated in keratinocytes, intestinal mucosa, brain, breast, and prostate tissues [11, 12]. Notably, lung tissue expresses low levels of 24-hydroxylase, the enzyme that inactivates 1,25-dihydroxyvitamin D, potentially permitting sustained activity once vitamin D is activated [10].

Extrapulmonary vitamin D activation has also been observed in other settings. For instance, a case report of a woman with chronic kidney disease who demonstrated increased 1,25-dihydroxyvitamin D during pregnancy highlights the role of the human placenta in expressing the 1α-hydroxylase enzyme [13]. In vitro studies of human fetal fibroblasts likewise showed expression of vitamin D receptor (VDR) and the enzymes involved in vitamin D metabolism CYP27A1, which metabolize vitamin D3 to 25-OH vitamin D, and CYP27B1, which converts 25-OH vitamin D to 1,25-dihydroxyvitamin D. These findings suggested that pulmonary fibroblasts can locally convert vitamin D to its active form [14].

Active vitamin D (1,25-dihydroxyvitamin D) plays a key role in regulating pulmonary inflammation through the induction of the antibacterial peptide cathelicidin [15]. In vitro, cathelicidin exhibits antibacterial, antiviral, and antifungal properties [16]. Cathelicidin also modulates the host immune response by inducing the production of cytokines and chemoattractants [17]. In the context of immune checkpoint therapy, upregulated immune activity could enhance extrarenal vitamin D activation, leading to excess cathelicidin production and immune amplification. This, in turn, may promote lung inflammation (pneumonitis) while concurrently driving 1,25-dihydroxyvitamin D–mediated hypercalcemia. Emerging evidence from the “*vitamin D cathelicidin axis*” supports the notion that downstream signals from vitamin D activation can enhance the immune system by encoding a series of genes with antimicrobial properties, including cathelicidin [18]. A similar phenomenon has been observed with MTX-induced 1,25-dihydroxyvitamin D–mediated hypercalcemia, paralleling pembrolizumab-induced hypercalcemia [19].

An alternative hypothesis involves granulomatous inflammation secondary to pembrolizumab-induced pulmonary hypersensitivity. Activated macrophages within granulomas express 1α-hydroxylase, leading to increased 1,25-dihydroxyvitamin D production and hypercalcemia [19]. While autopsy data is lacking for pembrolizumab-induced pneumonitis, insight can be drawn from MTX-induced pneumonitis, another immune-mediated entity associated with 1,25-dihydroxyvitamin D–driven hypercalcemia. In a review of 49 patients with MTX-induced pneumonitis, 34.7% were found to have granulomas on histological evaluation, with interstitial inflammation and fibrosis as the most common findings [17].

The degree of hypercalcemia seen in pembrolizumab-induced hypercalcemia can be severe, likely due to the unregulated nature of extrarenal 1α-hydroxylase. Unlike renal 1α-hydroxylase which is tightly regulated by PTH, fibroblast growth factor-23 (FGF-23), calcium, phosphorus, and negative feedback by 1,25-dihydroxyvitamin D, extrarenal enzyme activity such as in macrophages and prostate tissue is relatively resistant to these inhibitory mechanisms [20]. This lack of regulation likely contributes to the pronounced and persistent 1,25-dihydroxyvitamin D elevations observed in immune checkpoint inhibitor–related hypercalcemia [21].

Choosing the initial dose of corticosteroid for ICI-mediated hypercalcemia is based on expert opinion, degree of hypercalcemia, level of 1,25-dihydroxyvitamin D elevation, patient response to volume expansion, and patient comorbidities. In addition, some insight can be derived from gastroenterology and medical oncology societies regarding the management of ICI-mediated colitis. For grade 2 colitis (>4 bowel movements per day), these medical societies recommend temporarily stopping immunotherapy and using a dose of 1 mg/kg/day of prednisone or equivalent steroid then increasing the dose to 2 mg/kg/day if there is no symptomatic improvement in 7 days. With symptomatic improvement, steroids can be tapered for 4 to 6 weeks [22]. Following multidisciplinary discussion, if the risk-benefit ratio favors reinitiation of ICI, frequent lab monitoring, avoiding dehydration, and using other immunosuppressive agents such as calcineurin inhibitors can be tried, based on success in ICI-mediated colitis [23].

Pembrolizumab can rarely induce 1,25-dihydroxyvitamin D–mediated hypercalcemia, potentially through immune activation within the lung leading to extrarenal vitamin D activation or granulomatous inflammation. Recognition of this mechanism is critical, as management differs from humoral hypercalcemia of malignancy and requires discontinuation of the offending agent and immunosuppression with corticosteroids. Further studies and histopathologic analyses are warranted to elucidate the underlying pathways and inform targeted management strategies.

## Learning points

- Hypercalcemia in a patient with a history of malignancy can point to a grave prognosis. Awareness of immune checkpoint–mediated hypercalcemia can lead to early identification, diagnosis, and treatment.
- The differential diagnosis of PTH-independent hypercalcemia is broad, and a complete workup is necessary in all patients. In patients on ICI therapy, imaging of the lungs can help identify the association between pneumonitis and hypercalcemia.
- Hypercalcemia from ICI can develop at any time during the treatment with these agents.

## Contributors

All authors made individual contributions to authorship. N.B., A.H., and S.H. were directly involved in the care of one or both patients. V.B. was involved in authorship of case presentation and diagnostic assessment including creation of tables and organization of the manuscript. N.B. was involved in authorship of abstract and organization of the manuscript. H.W. was involved in authorship of the introduction and learning points. Z.W. was involved in authorship of treatment and outcome and follow-up. S.H. and A.H. were involved in authorship of discussion. All authors reviewed and approved the final draft.

## Data Availability

Data sharing is not applicable to this article and no datasets were generated or analyzed during this study.

## Abbreviations

CTLA-4: cytotoxic T-lymphocyte–associated antigen 4
ICI: immune checkpoint inhibitor
irAE: immune-related adverse event
MTX: methotrexate
PD-1: programmed cell death protein 1
PTH: parathyroid hormone
PTHrP: parathyroid hormone–related peptide
